# CircSPIDR acts as a tumour suppressor in cervical adenocarcinoma by sponging miR-431-5p and regulating SORCS1 and CUBN expression

**DOI:** 10.18632/aging.203283

**Published:** 2021-07-29

**Authors:** Junfen Xu, Weiguo Lu

**Affiliations:** 1 Department of Gynecologic Oncology, Women’s Hospital, Zhejiang University School of Medicine, Hangzhou 310006, Zhejiang, China; 2 Center of Uterine Cancer Diagnosis & Therapy of Zhejiang Province, Hangzhou 310006, Zhejiang, China; 3 Zhejiang University Cancer Center, Hangzhou 310006, Zhejiang, China

**Keywords:** CircSPIDR, cervical adenocarcinoma, miR-431-5p, SORCS1, CUBN, cell growth

## Abstract

To identify circular RNAs (circRNAs) with tumor suppressor activity against cervical adenocarcinoma, we compared the circRNA levels of cervical adenocarcinoma and normal cervical tissues. We found that circSPIDR was dramatically downregulated in cervical adenocarcinoma tissues. In cervical adenocarcinoma cells, overexpression of circSPIDR reduced cell viability, inhibited colony formation and promoted apoptosis, whereas knockdown of circSPIDR exerted the opposite effects. CircSPIDR overexpression also suppressed the tumorigenicity of cervical adenocarcinoma cells in a xenograft mouse model. CircSPIDR was found to sponge miR-431-5p, thereby de-repressing sortin-related VPS10 domain-containing receptor 1 (*SORCS1*) and cubilin (*CUBN*) and inhibiting the development of cervical adenocarcinoma. In clinical cervical samples, circSPIDR expression correlated negatively with miR-431-5p expression and positively with *SORCS1* and *CUBN* expression. These results demonstrated that circSPIDR suppresses cervical adenocarcinoma by competitively binding to miR-431-5p, thus upregulating *SORCS1* and *CUBN*. These findings suggest circSPIDR could serve as a novel therapeutic target for treatment of cervical adenocarcinoma patients.

## INTRODUCTION

Cervical cancer is the fourth most common cancer and the fourth leading cause of cancer death in women worldwide [1]. In China, approximately 98,900 new cervical cancer cases were diagnosed and 30,500 women died of this disease in 2015 [2]. Overall, cervical squamous cell carcinoma is the primary pathological type of human cervical cancer, while cervical adenocarcinoma (CADC) is the second. In recent decades, the tumor screenings and human papillomavirus vaccination programs have greatly reduced the burden of cervical squamous cell carcinoma in developed countries; however, the incidence of CADC has increased during the same period worldwide [2, 3]. Thus, it is critical to determine the underlying molecular mechanisms of CADC and develop effective therapies.

Circular RNAs (circRNAs) are regulatory RNAs characterized by covalent single-stranded loop structures generated via back-splicing or exon skipping of the precursor mRNA [4–7]. CircRNAs are naturally resistant to RNA exonucleases, are tissue- or cell type-specific [5, 8], and exert important functions in various human cancers [9, 10]. To date, circRNAs have largely been reported to serve as competing endogenous RNAs (ceRNAs). For example, circTP63 (an oncogene in lung squamous cell carcinoma), circ-ZKSCAN1 (a bladder cancer suppressor) and other cancer-related circRNAs such as CDR1as [11–13] and circAKT3 [14, 15] function as microRNA (miRNA) sponges that inhibit the effects of miRNAs on their target genes. In addition to the ceRNA mechanism, circRNAs can sequester specific RNA-binding proteins to regulate gene expression [4, 16], and some of them can be translated into functional proteins [17–19]. Certain circRNAs have been identified as promising clinical molecular biomarkers for cancer diagnosis and treatment [20–22]. However, the functions of circRNAs in CADC remain to be elucidated.

In the present study, our main interest was to identify circRNAs with tumor suppressor functions in CADC. In a previous study using RNA-sequencing data from human CADC and normal cervical tissues (GSE No. 145372), we found that one of the most significantly downregulated circRNAs in CADC tissues was circSPIDR, - a circRNA derived from exons 6 and 7 of the human scaffold protein involved in DNA repair (SPIDR) gene. Thus, in the current study, we evaluated the effects of circSPIDR on cell proliferation, colony formation and apoptosis in CADC cells. Then, we examined the influence of circSPIDR on miRNAs and their target genes, and determined the effects of these genes on the malignant behaviors of CADC cells. Finally, we assessed the clinical importance of circSPIDR expression by evaluating its correlation with miRNA and target gene expression in a large cohort of primary CADC tissues. Our study suggested that circSPIDR is a tumor suppressor that may be a candidate for the diagnosis and treatment of CADC.

## RESULTS

### CircSPIDR is significantly down-regulated in CADC

**Figure 1:**
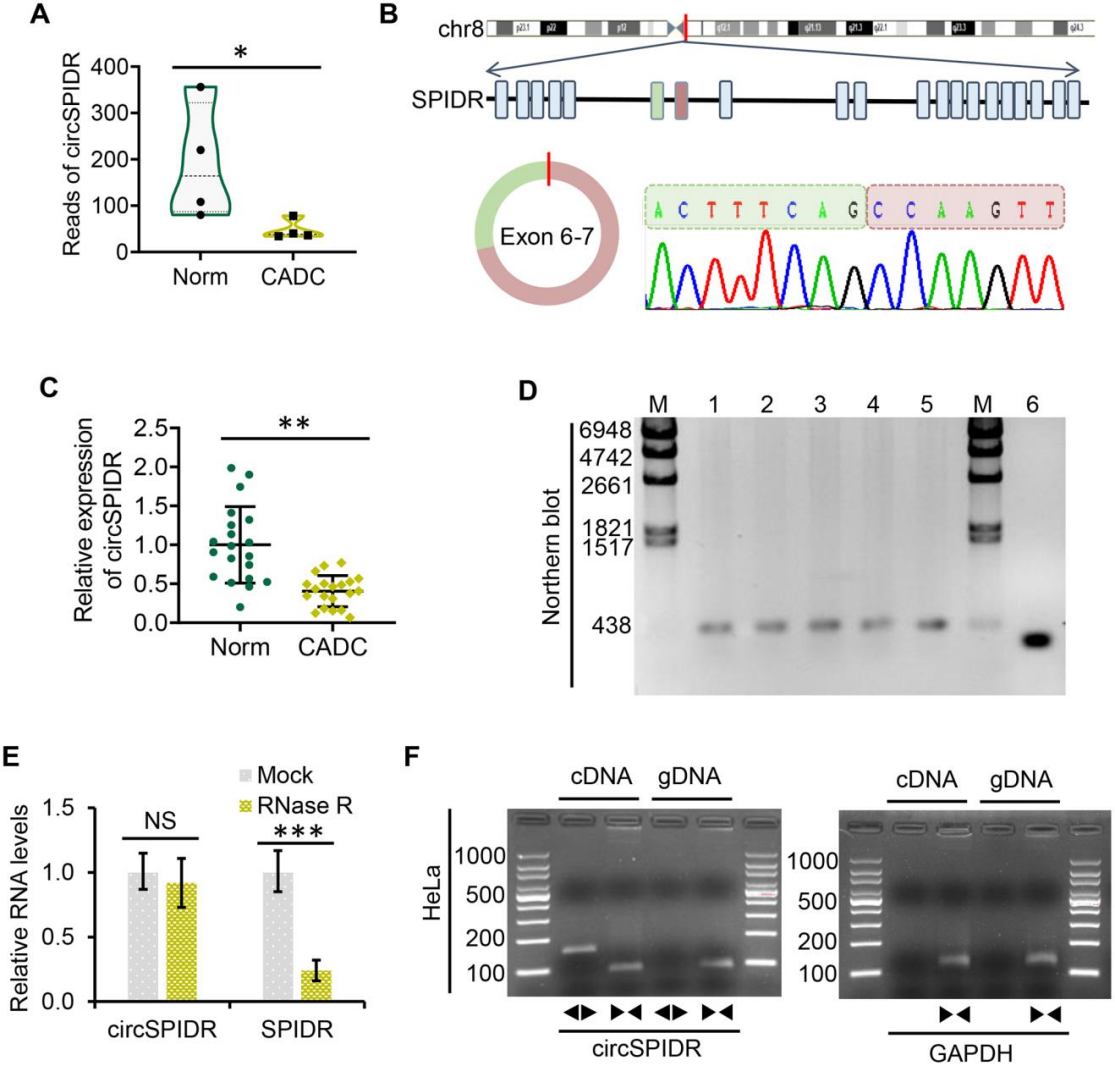
**Characterization and validation of circSPIDR expression in CADC.** (**A**) Violin plot showing the distribution of the RNA sequencing reads of circSPIDR in normal cervical tissues (Norm) and CADC tissues. (**B**) The genomic locus and generation of circSPIDR. CircSPIDR is produced from exons 6 and 7 of the human *SPIDR* gene. The back-splice junction sequence of circSPIDR was detected using Sanger sequencing. (**C**) qRT-PCR analysis of circSPIDR expression in 20 normal cervical tissues and 20 CADC tissues. (**D**) Northern blot of circSPIDR. Hybridization was performed with exon 6–7 junction probes. M, RNA marker; 1–3, HeLa cell repeats; 4, CADC tissue; 5, Normal cervical tissue; 6, RT-PCR products of probes. (**E**) qRT–PCR analysis of circSPIDR expression and *SPIDR* mRNA expression in HeLa cells with or without RNase R treatment. (**F**) RT-PCR products of circSPIDR and its linear isoform (*SPIDR)* in cDNA and gDNA from HeLa cells. *GAPDH* was used as a control. NS, not significant; ^*^P < 0.05; ^**^P < 0.01; ^***^P < 0.001.

We previously used transcriptome sequencing to determine the circRNA expression signatures of CADC patients [23], and found that a novel circRNA named circSPIDR was significantly downregulated in CADC tissues compared with normal cervical tissues (Figure 1A). CircSPIDR is derived from exons 6 and 7 of the human *SPIDR* gene, and is 352 nucleotides long (Figure 1B). To confirm our circRNA sequencing data, we used quantitative real-time PCR (qRT-PCR) to assess circSPIDR expression in another 40 human cervical samples (20 normal vs. 20 CADC tissues). CircSPIDR expression was much lower in CADC tissues than in normal control tissues (Figure 1C).

### Characterization of circSPIDR in CADC cells

Next, we used Sanger sequencing to assess the structure of circSPIDR (Figure 1B). Northern blotting confirmed that circSPIDR could be detected at approximately 352 nucleotides with a probe targeting the back-spliced junction in CADC cells (HeLa) and tissues (Figure 1D). A stability analysis of circSPIDR and *SPIDR* indicated that the loop structure of circSPIDR was resistant to digestion with RNase R exonuclease, while the linear *SPIDR* mRNA was degraded upon RNase R treatment (Figure 1E).

To determine whether head-to-tail splicing was the result of trans-splicing or genomic rearrangement, we designed divergent and convergent primers for circSPIDR. An RT-PCR analysis of reverse-transcribed RNA (cDNA) and genomic DNA (gDNA) from HeLa cells indicated that the divergent circSPIDR primers could amplify products from the cDNA, but not from the gDNA (Figure 1F). These results confirmed that circSPIDR is a stable circRNA expressed in CADC cells.

### CircSPIDR exerts tumor-suppressive effects in CADC cells *in vitro* and *in vivo*

To evaluate the effects of circSPIDR on CADC cells, we conducted a circSPIDR expression vector and two specific siRNAs against circSPIDR. The overexpression plasmids and siRNAs were transfected into HeLa cells, and the cells were harvested for experiments after 48 h. The circSPIDR vector successfully increased circSPIDR expression rather than SPIDR mRNA expression in HeLa cells (Supplementary Figure 1A). CircSPIDR siRNA #1 and #2 specifically inhibited circSPIDR expression without influencing *SPIDR* mRNA expression (Supplementary Figure 1B). These results also indicated that *SPIDR* expression was not altered by circSPIDR expression.

**Figure 2:**
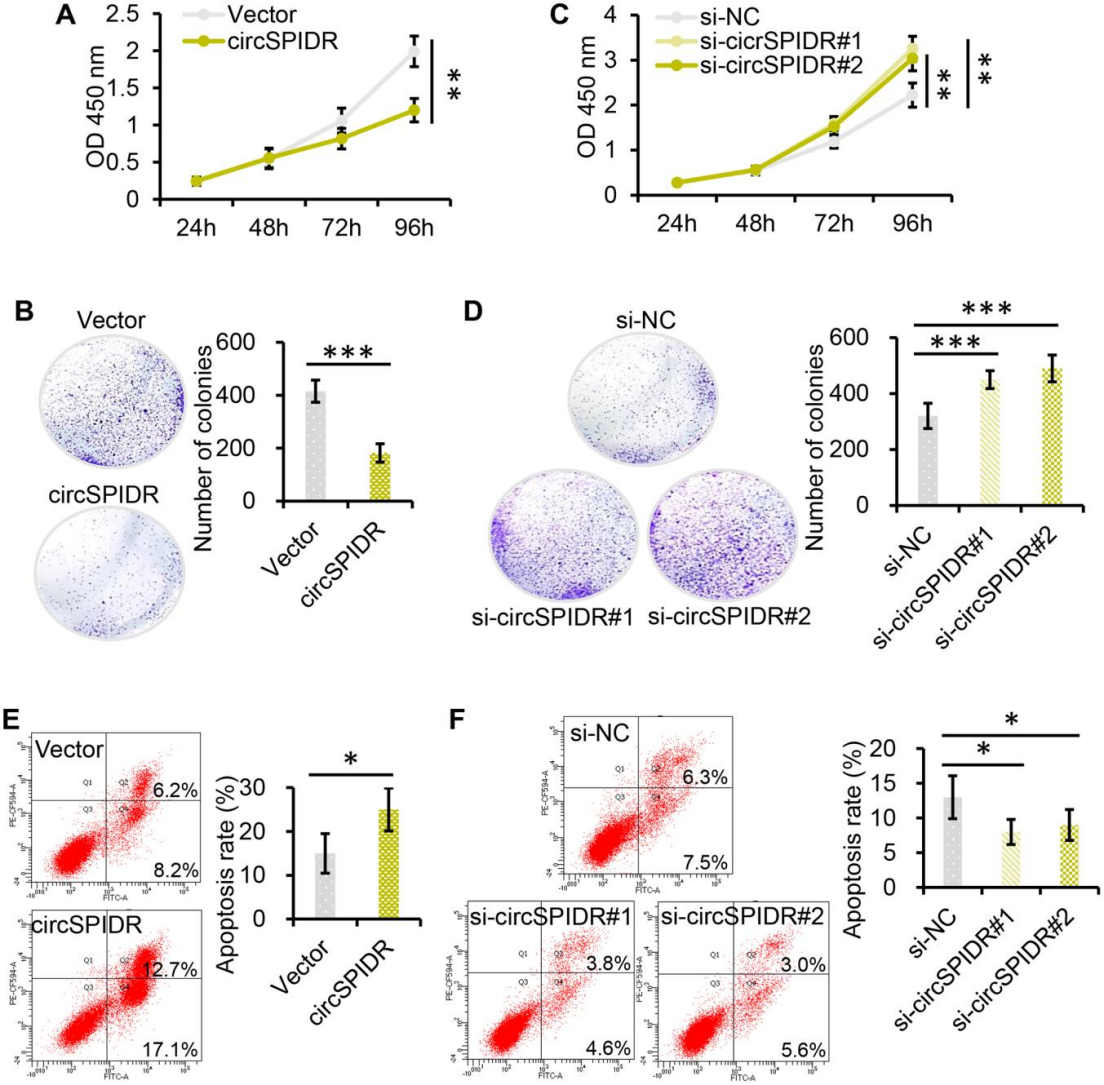
**CircSPIDR inhibits cell growth and promotes apoptosis in CADC cells *in vitro*.** (**A**, **C**) CCK-8 assays demonstrated that circSPIDR overexpression reduced HeLa cell viability (**A**), while circSPIDR inhibition with specific siRNAs (si-circSPIDR#1 or si-circSPIDR#2) increased HeLa cell viability (**C**). (**B**, **D**) Colony formation assays indicated that circSPIDR overexpression suppressed colony formation in HeLa cells (**B**), while circSPIDR inhibition promoted colony formation (**D**). (**E**, **F**) Representative images from flow cytometry analysis of apoptosis. Apoptotic cells were determined using Annexin V-FITC (X-axis) and PI (Y-axis) staining. CircSPIDR overexpression promoted HeLa cell apoptosis, while circSPIDR inhibition had the opposite effect. ^*^P < 0.05; ^**^P < 0.01; ^***^P < 0.001.

We then performed Cell Counting Kit 8 (CCK-8) and colony formation assays. Which demonstrated that overexpression of circSPIDR significantly suppressed HeLa cell viability and colony formation (Figure 2A and 2B). Conversely, knockdown of circSPIDR significantly enhanced cell viability and colony formation (Figure 2C and 2D). A bromodeoxyuridine (BrdU) incorporation assay indicated that the percentage of the BrdU-positive (proliferating) cells was lower in circSPIDR-overexpressing HeLa cells than in vector control cells (Supplementary Figure 2). Apoptosis assays illustrated that circSPIDR overexpression remarkably increased the proportion of apoptotic HeLa cells (Figure 2E), whereas circSPIDR knockdown had the opposite effect (Figure 2F). These results indicated that circSPIDR significantly suppressed cell proliferation and induced apoptosis in HeLa cells.

**Figure 3:**
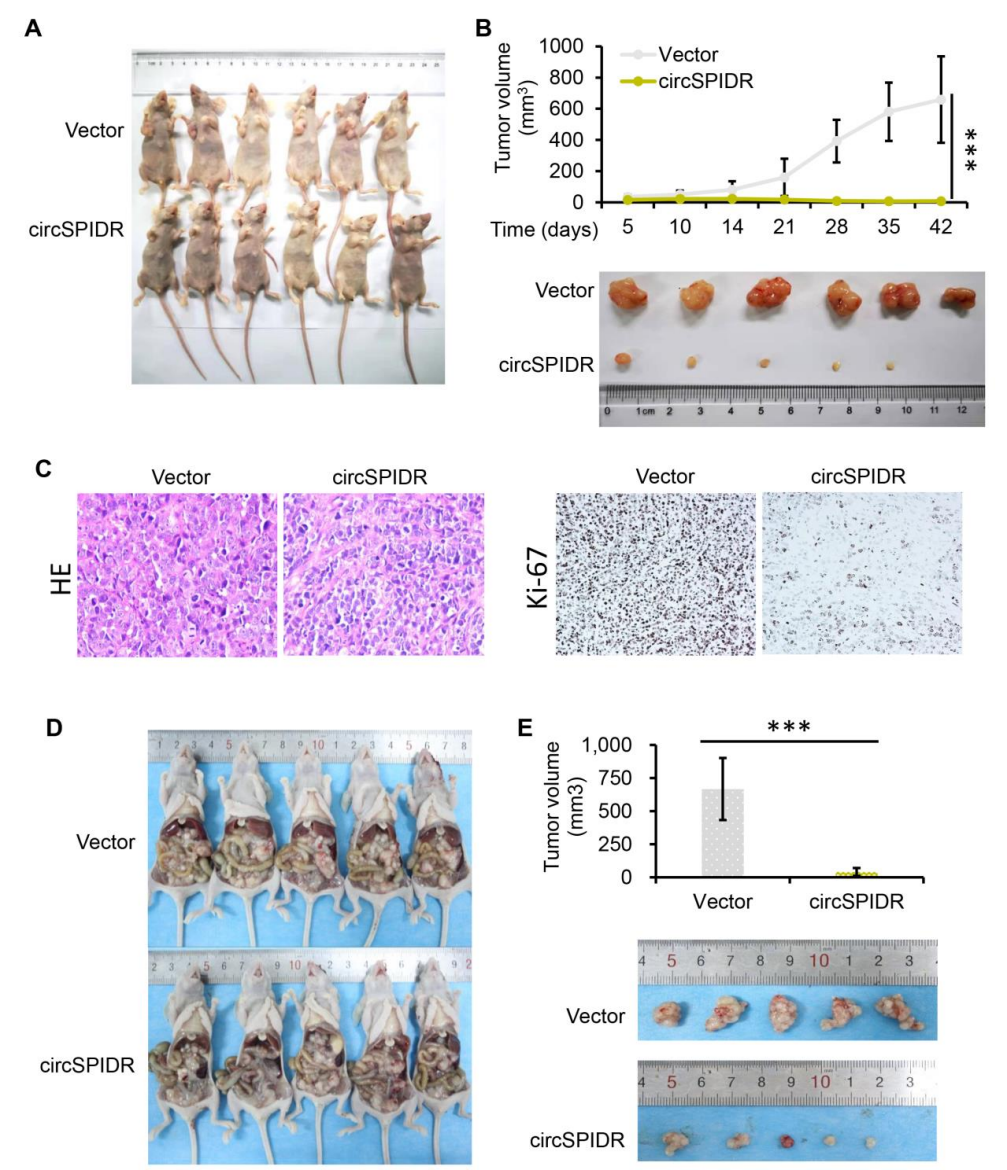
**CircSPIDR overexpression inhibits HeLa cell growth *in vivo*.** (**A**) HeLa cells expressing circSPIDR or the vector control were inoculated into BALB/c nude mice (*n* = 6/group) to establish subcutaneous xenograft tumors. Representative images of nude mice bearing CADC tumors are shown. (**B**) Growth curves and representative images of isolated xenograft tumours. The volume of the local tumors was measured. (**C**) Hematoxylin and eosin staining was performed and Ki-67 protein expression was evaluated in the xenograft tumors. (**D**, **E**) Representative images of nude mice intraperitoneally transplanted with HeLa/circSPIDR and HeLa/vector cells. ^***^P < 0.001.

To identify the effects of circSPIDR *in vivo*, we stably expressed circSPIDR or an empty vector in HeLa cells using neomycin selection for 10 days. Then, we subcutaneously injected these cells into the right single flank of BALB/c nude mice (*n* = 6/group) to establish a xenograft tumor model. The tumors formed after one week, and the tumor size were monitored for the next five weeks. Tumors derived from circSPIDR overexpressing cells were much smaller than those derived from control cells (Figure 3A and 3B), suggesting that circSPIDR significantly inhibited tumor growth. Immunohistochemical staining of the xenograft tumor tissues demonstrated that the proliferation marker Ki-67 was expressed at lower levels in the circSPIDR-overexpressing group (Figure 3C). In addition to generating the subcutaneous tumor model, we also intraperitoneally injected the two groups of cells into nude mice (*n* = 5/group), and found that the tumors were much smaller in the circSPIDR group than in the vector control group (Figure 3D and 3E). These results suggested that circSPIDR suppressed CADC tumor growth *in vitro* and *in vivo*.

### CircSPIDR sponges miR-431-5p in CADC cells

One major function of circRNAs is to sponge miRNAs [24]. To identify miRNAs that could be targets of circSPIDR, we searched the miRanda bioinformatics prediction database. Then, we compared the results with the differentially expressed miRNA signature of CADC patients from our previous miRNA sequencing data [25]. We found that circSPIDR possessed the conserved target site of miR-431-5p, and that miR-431-5p was upregulated in CADC tissues.

**Figure 4:**
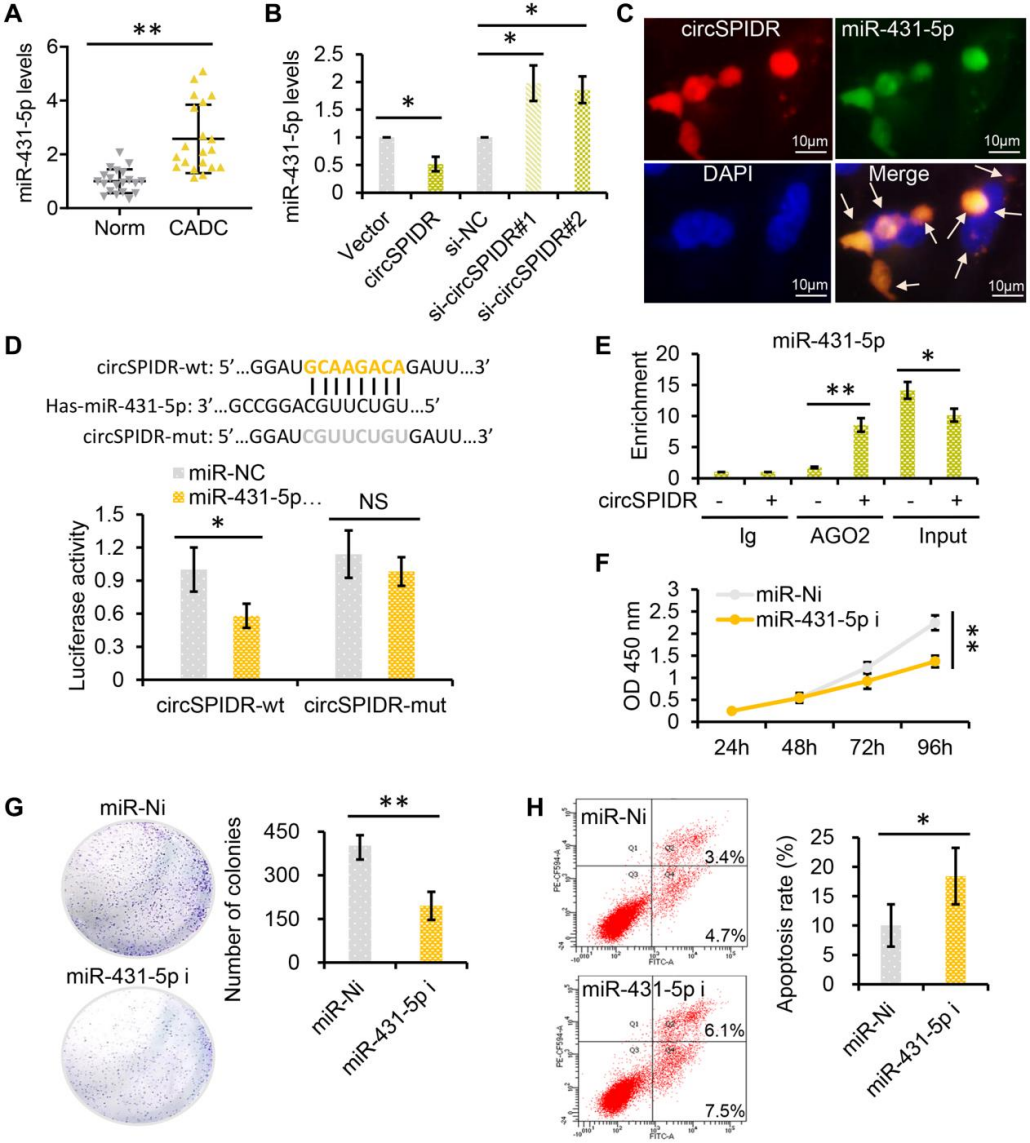
**CircSPIDR directly binds to miR-431-5p as a miRNA sponge.** (**A**) qRT-PCR analysis of miR-431-5p expression in 20 normal cervical tissues and 20 CADC tissues. (**B**) qRT-PCR analysis of miR-431-5p expression in HeLa cells transfected with circSPIDR, the vector, si-circSPIDR#1, si-circSPIDR#2 or si-NC. (**C**) RNA fluorescence *in situ* hybridization assay for circSPIDR and miR-431-5p in HeLa cells. (**D**) Schematic drawing showing the putative binding sites of miR-431-5p on circSPIDR. A luciferase reporter assay was performed to detect circSPIDR luciferase reporter activity in cells co-transfected with miR-431-5p mimics or miR-NC. (**E**) AGO2-RIP was conducted using an anti-AGO2 antibody in HeLa cells transfected with circSPIDR or the vector. The enrichment of miR-431-5p was then assessed using qRT-PCR. (**F**–**G**) CCK-8 (**F**) and colony formation (**G**) assays of HeLa cells transfected with miR-431-5p inhibitors. (**H**) Apoptosis analysis of HeLa cells transfected with miR-431-5p inhibitors. NS, not significant; ^*^P < 0.05; ^**^P < 0.01.

To test the hypothesis that circSPIDR sponges miR-431-5p, we first examined miR-431-5p levels in the same 40 human cervical samples assayed for circSPIDR expression above. The qRT-PCR results revealed that miR-431-5p expression was significantly greater in CADC tissues with lower circSPIDR expression (Figure 4A). Next, we assessed the effects of circSPIDR expression on miR-431-5p expression in HeLa cells, and found that circSPIDR overexpression significantly reduced miR-431-5p expression, while circSPIDR knockdown increased miR-431-5p expression (Figure 4B). We then performed an RNA fluorescence *in situ* hybridization assay, which demonstrated that circSPIDR co-localized with miR-431-5p in HeLa cells (Figure 4C). Of note, circSPIDR and miR-431-5p were localized in both the cytoplasm and the nucleus, indicating that these RNAs may have additional functions aside from serving as ceRNAs.

Subsequently, we performed a dual luciferase reporter assay in 293T cells transfected with miR-431-5p mimics and luciferase vectors fused with circSPIDR wild-type (wt) or mutant (mut) promoters. As shown in Figure 4D, when miR-431-5p mimics were present, luciferase reporter activity was significantly reduced in the circSPIDR-wt group, but not in the circSPIDR-mut group. In addition, we performed an RNA immunoprecipitation for argonaute 2 (AGO2-RIP) assay in circSPIDR-overexpressing and control HeLa cells. The results revealed that circSPIDR and miR-431-5p were substantially enriched by AGO2 (Figure 4E). These findings indicated that circSPIDR could bind directly to miR-431-5p.

To explore the function of miR-431-5p in CADC cells, we treated HeLa cells with inhibitors of miR-431-5p. CCK-8 and colony formation assays demonstrated that miR-431-5p inhibitors significantly reduced the growth of HeLa cells (Figure 4F and 4G). In addition, miR-431-5p inhibitors promoted Hela cell apoptosis (Figure 4H). Thus, miR-431-5p inhibition mimicked the phenotypic effects of circSPIDR overexpression in HeLa cells.

**Figure 5:**
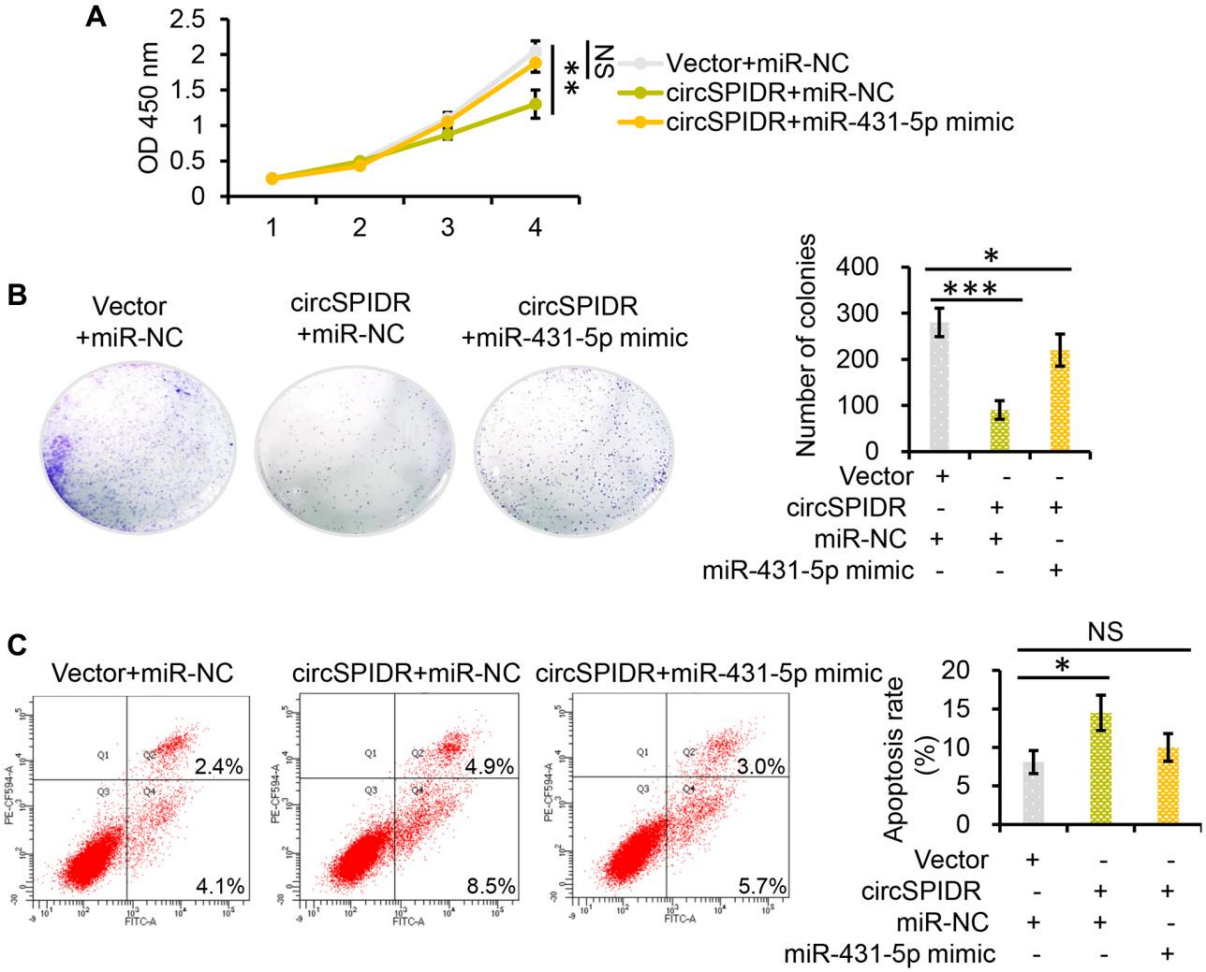
**MiR-431-5p reverses the tumor-suppressive effects of circSPIDR in CADC cells.** (**A**) CCK-8 assay evaluating the viability of HeLa cells co-transfected with circSPIDR and miR-431-5p mimics. (**B**) Colony formation assay in HeLa cells co-transfected with circSPIDR and miR-431-5p mimics. (**C**) Apoptosis analysis of HeLa cells co-transfected with circSPIDR and miR-431-5p mimics. NS, not significant; ^*^P < 0.05; ^**^P < 0.01; ^***^P < 0.001.

Next, we used miR-431-5p mimics to examine whether miR-431-5p overexpression could nullify the effects of circSPIDR overexpression in CADC cells. In rescue experiments, miR-431-5p overexpression reversed the circSPIDR-induced suppression of CADC cell proliferation and colony formation (Figure 5A and 5B). Co-transfection of circSPIDR with the miR-431-5p negative control (miR-NC) induced apoptosis in CADC cells, consistent with the effects of circSPIDR alone (see Figure 2E); however, miR-431-5p mimics impaired the apoptotic effects of circSPIDR (Figure 5C). These results demonstrated that miR-431-5p is the critical target of circSPIDR sponging activity in CADC.

### CircSPIDR relieves the repression of *SORCS1* and *CUBN* by miR-431-5p in CADC cells

**Figure 6:**
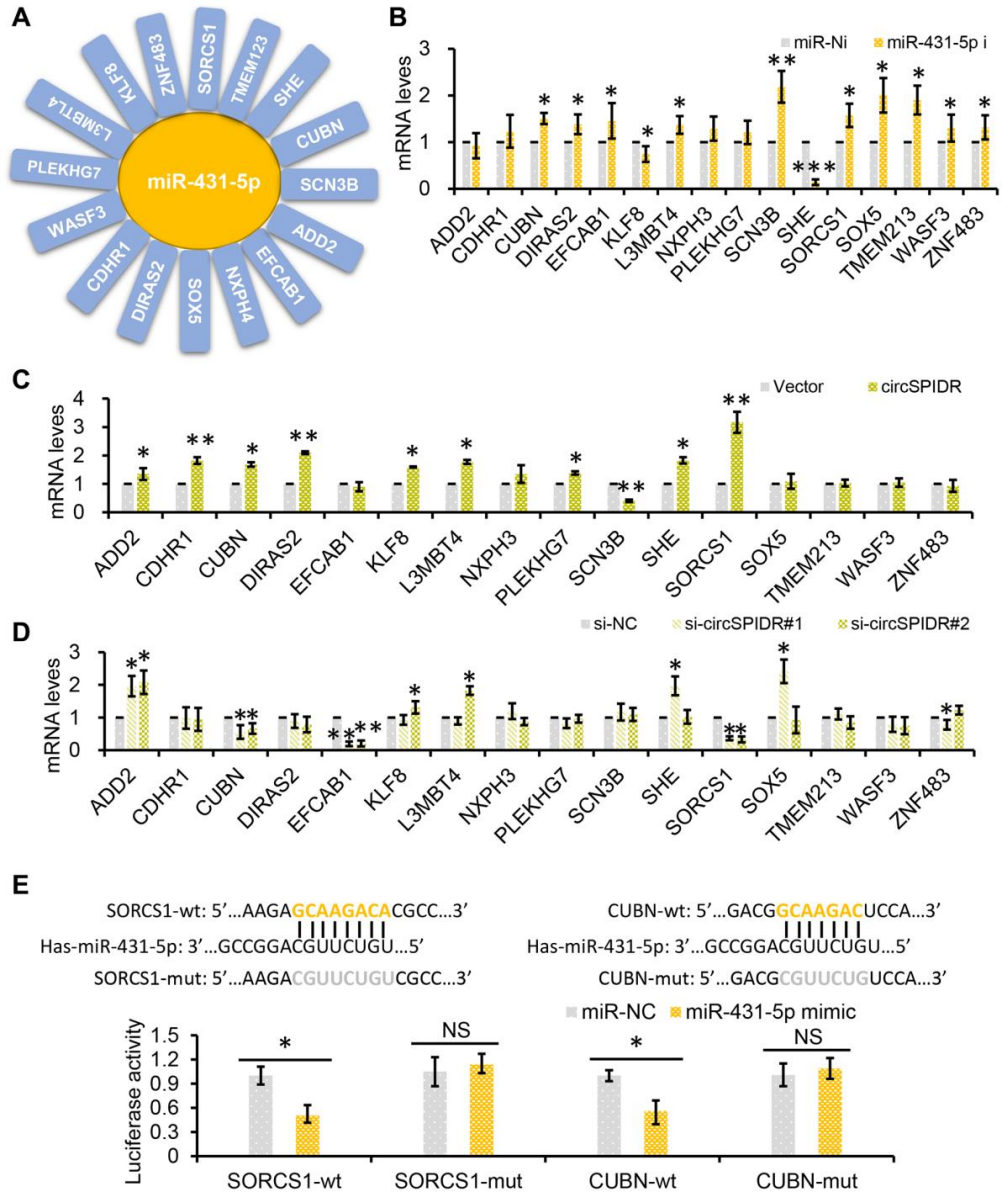
***SORCS1* and *CUBN* are the target genes of miR-431-5p and circSPIDR.** (**A**) miR-431-5p-target gene regulatory network. (**B**) qRT-PCR analysis of candidate target gene expression in CADC cells transfected with miR-431-5p inhibitors. (**C**, **D**) qRT-PCR analysis of candidate target gene expression in CADC cells transfected with the circSPIDR expression vector (**C**) or siRNAs against circSPIDR (**D**). (**E**) Luciferase activity of the 3′UTRs of *SORCS1* and the *CUBN* in 293T cells co-transfected with miR-431-5p mimics. NS, not significant; ^*^P < 0.05; ^**^P < 0.01.

Based on the ceRNA theory, we hypothesized that circSPIDR could enhance the expression of miR-431-5p target genes by sponging miR-431-5p. Using TargetScan and miRanda, we filtered the potential direct targets of miR-431-5p and mapped them to our previous transcriptome sequencing data for CADC [25]. We thus identified 16 putative miR-431-5p target genes that were significantly downregulated in CADC tissues: *ADD2, CDHR1, CUBN, DIRAS2, EFCAB1, KLF8, L3MBT4, NXPH3, PLEKHG7, SCN3B, SHE, SORCS1, SOX5, TMEM213, WASF3 and ZNF483.* (Figure 6A).

Next, we used miR-431-5p inhibitors to examine the effects of miR-431-5p on the expression of these genes in HeLa cells. Treatment of CADC cells with miR-431-5p inhibitors strongly increased the expression of *CUBN, DIRAS2, EFCAB1, L3MBT4, SCN3B, SORCS1, SOX5, TMEM213, WASF3 and ZNF483*, but not the other genes (Figure 6B). However, overexpression of circSPIDR in HeLa cells only significantly upregulate *CUBN* (cubilin) and *SORCS1* (sortilin-related VPS10 domain-containing receptor 1), and knockdown of circSPIDR only significantly downregulated the same two genes (Figure 6C and 6D).

**Figure 7:**
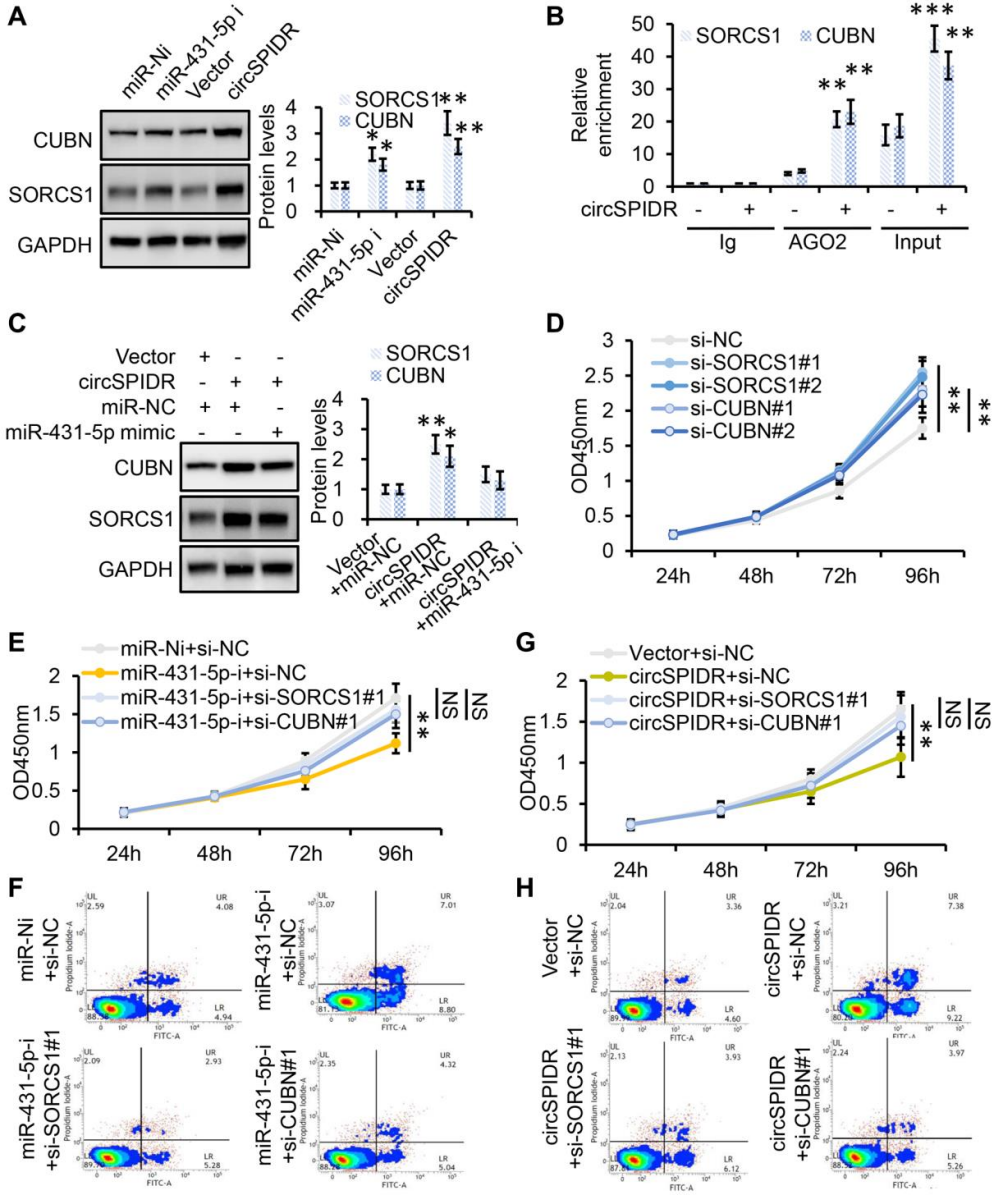
***SORCS1* and *CUBN* are the functional targets of circSPIDR/miR-431-5p signaling.** (**A**) Western blot analysis of SORCS1 and CUBN protein levels in CADC cells transfected with miR-431-5p inhibitors or the circSPIDR expression vector. (**B**) AGO2-RIP was performed using an anti-AGO2 antibody in HeLa cells transfected with circSPIDR or the vector. Then, qRT-PCR was used to assess and the enrichment of *SORCS1* and *CUBN*. (**C**) Western blot analysis of SORCS1 and CUBN protein expression in CADC cells co-transfected with circSPIDR and miR-431-5p mimics. (**D**) CCK-8 assay in *SORCS1-* or *CUBN*-knockdown HeLa cells. (**E**, **F**) CCK-8 assay (**E**) and apoptosis assay (**F**) in HeLa cells following miR-431-5p inhibition and *SORCS1* or *CUBN* knockdown. (**G**, **H**) CCK-8 assay (**G**) and apoptosis assay (**H**) in HeLa cells following circSPIDR overexpression and *SORCS1* or *CUBN* knockdown. NS, not significant; ^*^P < 0.05; ^**^P < 0.01; ^***^P < 0.001.

To verify that *SORCS1* and *CUBN* were the bona fide targets of miR-431-5p, we constructed *SORCS1* 3′ untranslated region (UTR)-wt, *SORCS1* 3′UTR-mut, *CUBN* 3′UTR-wt, and *CUBN* 3′UTR-mut luciferase reporter systems and co-transfected them with miR-431-5p mimic into 293T cells. The miR-431-5p mimics significantly reduced the activities of the luciferase reporter vectors carrying *SORCS1* 3′UTR-wt or *CUBN* 3′UTR-wt sequences, but not of the vectors containing *SORCS1* 3′UTR- mut or *CUBN* 3′UTR- mut sequences (Figure 6E). Furthermore, inhibition of miR-431-5p markedly increased the protein levels of SORCS1 and CUBN in CADC cells (Figure 7A). Consistently, overexpression of circSPIDR significantly increased the protein levels of SORCS1 and CUBN in HeLa cells (Figure 7A).

To test whether *SORCS1* and *CUBN* were localized with the circSPIDR/miR-431-5p sponge complex, we performed an AGO2-RIP assay, which indicated that circSPIDR, miR-431-5p, *SORCS1* and *CUBN* were mainly enriched with AGO2 (Figures 4E, 7B). In addition, the up-regulation of SORCS1 and CUBN in circSPIDR-overexpressing CADC cells could be partially reversed by co-transfection with miR-431-5p mimics (Figure 7C). Therefore, we considered *SORCS1* and *CUBN* as the major downstream targets of miR-431-5p and circSPIDR.

Next, we assessed the involvement of SORCS1 and CUBN in circSPIDR/miR-431-5p signaling. The knockdown of *SORCS1* or *CUBN* using specific siRNAs significantly promoted cell survival and reduced apoptosis in CADC cells (Figure 7D, Supplementary Figure 3A and 3B). Moreover, *SORCS1* or *CUBN* knockdown partially reversed the proliferation-suppressive effects (Figure 7E) and abrogated the pro-apoptotic effects (Figure 7F, Supplementary Figure 4A) of miR-431-5p inhibitors. *SORCS1* and *CUBN* siRNAs reversed the suppression of cell proliferation (Figure 7G) and partially reversed the induction of apoptosis in circSPIDR-overexpressing HeLa cells (Figure 7H, Supplementary Figure 4B). These results suggested that *SORCS1* and *CUBN* are direct functional targets of circSPIDR/miR-431-5p signalling.

### The clinical value of circSPIDR in human cervical samples

**Figure 8:**
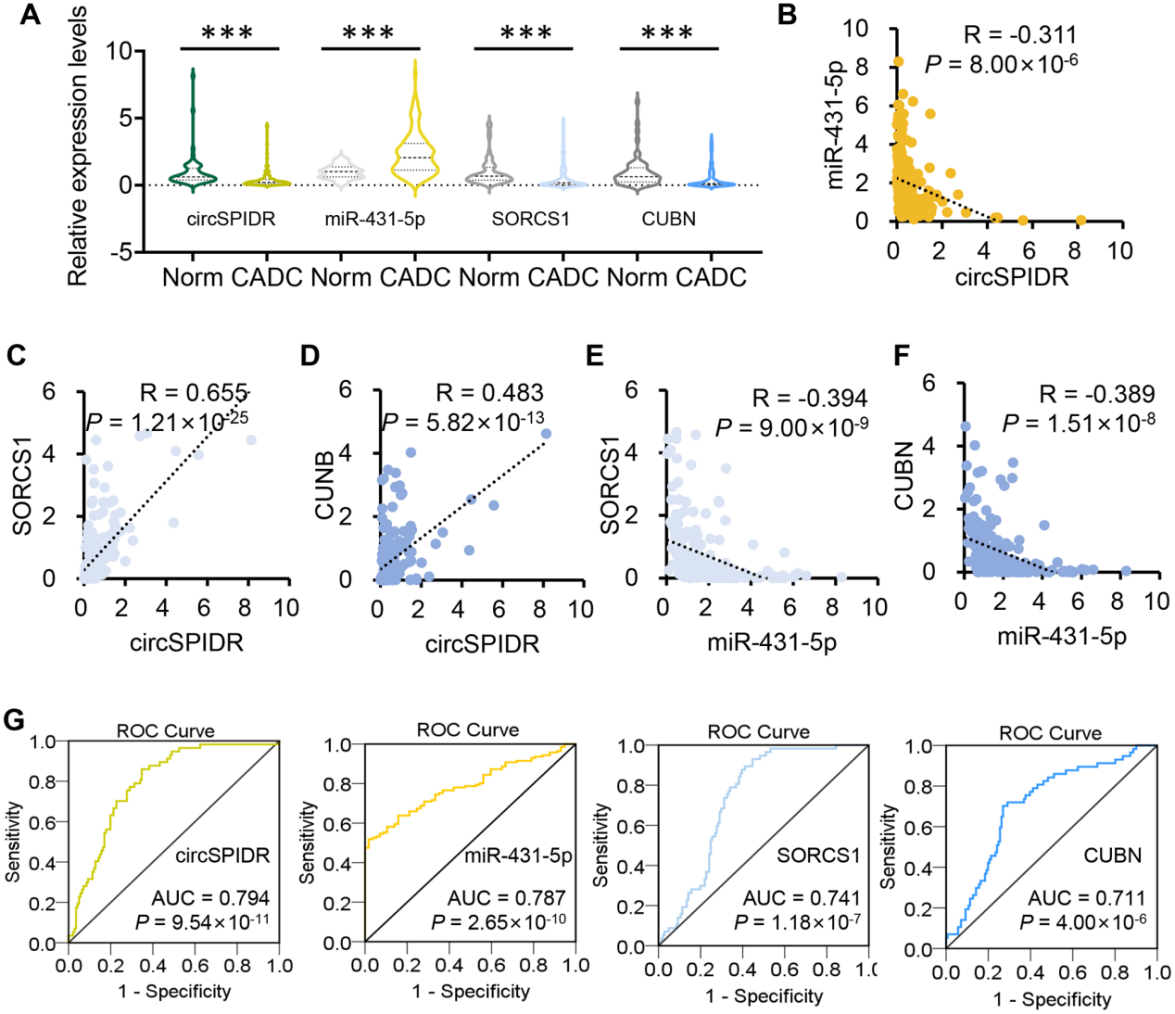
**The clinical value of circSPIDR, miR-431-5p, *SORCS1* and *CUBN* expression in CADC tissues.** (**A**) Violin plots showing circSPIDR, miR-431-5p, *SORCS1* and CUBN levels determined using qRT-PCR in 57 normal cervical tissues and 141 CADC tissues. (**B**–**D**) Pearson correlation analysis between circSPIDR levels and miR-431-5p (**B**), *SORCS1* (**C**), *CUBN* (**D**) levels. (**E**, **F**) Pearson correlation analysis between miR-431-5p levels and *SORCS1* (**E**) or *CUBN* (**F**) levels. (**G**) ROC curve analysis of circSPIDR, miR-431-5p, *SORCS1* and *CUBN* detection for CADC diagnosis. ^***^P < 0.001.

To explore the clinical value of circSPIDR, we collected an additional 57 normal human cervical tissues and 141 CADC tissues. A qRT-PCR analysis demonstrated that circSPIDR, *CUBN* and *SORCS1* were significantly down-regulated in CADC tissues compared with normal cervical tissues, while miR-431-5p was remarkably upregulated in CADC tissues (Figure 8A). To determine the correlation among these candidate biomarkers, we performed a Pearson’s correlation analysis. CircSPIDR levels exhibited a significant inverse correlation with miR-431-5p levels (Figure 8B), but exhibited significant positive correlations with *SORCS1* (Figure 8C) and *CUBN* levels (Figure 8D). The expression of miR-431-5p correlated inversely with the levels of *SORCS1* (Figure 8E) and *CUBN* (Figure 8F).

In addition, we performed a receiver operating characteristic (ROC) curve analysis to determine their diagnostic value of these genes in identifying CADC. The area under the ROC curve (AUC, representing the average sensitivity for all possible specificity values) was 0.794 for circSPIDR, 0.787 for miR-431-5p, 0.741 for *SORCS1* and 0.711 for *CUBN*, where an AUC > 0.5 is considered significant (Figure 8G). These data revealed that circSPIDR, miR-431-5p, *SORCS1* and *CUBN* have good potential as diagnostic markers for CADC.

## DISCUSSION

Numerous circRNAs serve as ceRNAs that bind to specific miRNAs and upregualte their target genes, thus promoting tumor development and progression [26–28]. For example, the well-known circRNA CDR1a competitively binds to miR-7 to promote the progression of many cancer types [11, 29–31]. Likewise, circTADA2A sponges miR-203a-3p, thus disinhibiting cyclic adenosine monophosphate responsive element binding protein 3 (CREB3) and increasing the malignant behavior of osteosarcoma [32]. We previously used RNA-sequencing to compare the circRNA, miRNA and mRNA signatures of CADC and normal cervical tissues, in order to identify potential oncogenic and tumor-suppressive circRNAs in CADC [23, 25]. We proposed that circEYA1 is a ceRNA that binds directly to miR-582-3p to enhance the expression of the miR-582-3p target gene C-X-C motif chemokine ligand 14 (CXCL14) [23]. In the same study, we also identified circSPIDR as a significantly downregulated circRNA in CADC tissues.

CircSPIDR is a novel human circRNA, so its functions had not previously been elucidated. Rat circSPIDR has been reported to enhance axon regeneration after sciatic nerve injury partially by altering the phosphoinositide 3-kinase/AKT pathway in dorsal root ganglions [33]. In the present study, we confirmed that circSPIDR was significantly downregulated in CADC tissues. Overexpression of circSPIDR reduced cell viability, inhibited colony formation and promoted apoptosis in CADC cells, while knockdown of circSPIDR had the opposite effects. CircSPIDR suppressed CADC cell growth both *in vitro* and *in vivo*, suggesting that this circRNA is a tumor suppressor in CADC.

To explore the potential ceRNA mechanism of circSPIDR, we performed bioinformatic analyses, which indicated that miR-431-5p shares a binding site with circSPIDR. MiR-431-5p has not been well studied, although it has been proposed as a tumor suppressor in certain cancer types. For instance, miR-431-5p was found to be down-regulated in colon cancer [34] and lung cancer [35], and circ_0001742 was reported to promote tongue squamous cell carcinoma by suppressing miR-431-5p, thus de-repressing activating transcription factor 3 (ATF3) [36]. However, our experiments revealed that, miR-431-5p was significantly up-regulated in CADC tissues, promoted CADC cell growth and reversed the tumor-suppressive effects of circSPIDR in HeLa cells. Luciferase reporter assays indicated that miR-431-5p reduced the activity of the circSPIDR-wt luciferase reporter, and AGO2-RIP analyses verified that circSPIDR could bind to miR-431-5p. These results suggested that circSPIDR bind to miR-431-5p to suppress CADC tumor growth.

We next searched for direct targets of miR-431-5p in HeLa cells, and identified *SORCS1* and *CUBN*. SORCS1 is a sorting-related receptor that is involved in metabolic control [37] and has been associated with diabetes in mice and humans [38–41]. *SORCS1* was found to be hypermethylated in colorectal cancer tissues, and reduced SORCS1 expression was identified as an independent prognostic factor in colorectal cancer patients [42]. CUBN is an intrinsic factor-vitamin B12 receptor, and mutation of *CUBN* was found to cause megaloblastic anaemia 1 [43]. Positive CUBN expression was associated with a better prognosis in clear cell renal cell carcinoma patients [44]. Here, we found that *SORCS1* and *CUBN* were down-regulated in CADC tissues, negatively regulated by miR-431-5p and positively regulated by circSPIDR. Correlation analyses indicated that circSPIDR, miR-431-5p, *SORCS1* and *CUBN* levels were tightly correlated in CADC tissues. Moreover, ROC curve analyses suggested that circSPIDR, miR-431-5p, *SORCS1* and *CUBN* expression had good diagnostic potential in CADC patients.

One limitation of our study was that we only used HeLa cells, the currently available CADC cell line. However, we confirmed our observations both in a large cohort of clinical CADC tissues and in animal models, and obtained consistent results. Overall, our findings demonstrated that circSPIDR was significantly down-regulated in CADC tissues and could suppress CADC cell growth by competitively binding to miR-431-5p, thus de-repressing *SORCS1* and *CUBN*. Our study has provided insight into circRNA activity in CADC and revealed the potential of circSPIDR as a therapeutic target in this disease.

## MATERIALS AND METHODS

### Human tissue specimen collection and ethical approval

This study was approved by the Ethics Committee of Women’s Hospital, Zhejiang University School of Medicine (IRB-2019062-R). Written informed consent was obtained from the patients. The study was performed in accordance with the Declaration of Helsinki. In total, 77 normal cervical tissues and 161 CADC tissues were obtained from Women’s Hospital, Zhejiang University School of Medicine from 2009 to 2019. Among them, 40 samples (20 normal cervical tissues and 20 CADC tissues) were used for the initial analysis of circSPIDR and miR-431-5p expression, and while the remaining 198 samples were used for qRT-PCR verification of all the candidate genes.

### Cell culture

The human CADC HeLa cell line was purchased from the Institute of Biochemistry and Cell Biology of the Chinese Academy of Sciences (Shanghai, China) and tested negative for mycoplasma contamination. HeLa cells were cultured in Minimum Essential Medium (Cellmax) with 10% fetal bovine serum (Sijiqing, China).

### Plasmids, siRNAs, miRNA mimics and miRNA inhibitors

The circSPIDR expression plasmid, the siRNAs targeting circSPIDR, *SORCS1* and *CUBN*, the miR-431-5p mimics, the miR-431-5p inhibitors and the relevant negative controls (pEX-3 empty vector, si-NC, miR-NC, and miR-Ni) were all purchased from GenePharma (Shanghai, China). The circSPIDR overexpression plasmids and siRNAs were transfected into HeLa cells using X-tremeGENE HP DNA transfection reagent (Roche, USA) and DharmaFECT1 transfection reagent (Dharmacon), respectively. The transfection experiments were conducted as described previously [23].

### RNA and gDNA extraction, RNase R treatment, RT-PCR, and qRT-PCR

The RNA and gDNA extraction, RNase R treatment, RT-PCR, and qRT-PCR were performed as previously reported [23]. The primers used in this study are listed in Supplementary Table 1.

### Northern blotting

Total RNA was extracted from the indicated cells and tissues using TRIzol (Invitrogen) and digested with RNase R. Subsequently, a 15-μg sample was loaded onto a 1% formaldehyde agarose gel and transferred to a Hybond N+ membrane (Amersham) via capillary transfer. Digoxigenin-deoxyuridine triphosphate (DIG-dUTP)-labeled circSPIDR probes were generated using a PCR DIG Probe Synthesis Kit (Roche) with the following primers: 5′-CCACAGCTAAGTTTCCCAGGAC-3′ and 5′-TGAGGTGTATGCAAAATGGTCT-3′. The PCR products were gel purified and sequenced prior to hybridization. Hybridization was performed with dUTP-labelled circSPIDR probes in DIG Easy Hyb buffer at 50°C overnight. The membrane was then washed and detected in accordance with the instructions for the DIG High Prime DNA Labeling and Detection Starter Kit II (Roche). Finally, the membrane was exposed to X-ray film for 10 min.

### CCK-8 and colony formation assays

CCK-8 and colony formation assays were performed as previously reported [23].

### Apoptosis assay

Treated cells were resuspended in binding buffer containing Annexin V-fluorescein isothiocyanate (FITC) and propidium iodide (PI) for 15 min in the dark (MultiScientces, China). The apoptotic rate was detected with a FACSVerse or FACSCalibur flow cytometer (BD Biosciences).

### BrdU assay

On the basis of the specifications of BrdU (Sigma), HeLa cell proliferation was assessed using a FITC-BrdU Cell Proliferation Detection Kit (KGA319-1, KeyGen Biotech, Nanjing, China). In brief, HeLa cells were incubated in a six-well plate and transfected with siRNAs or plasmids. After 48 h of transfection, 30 μM BrdU was added and incubated with the cells for 2 h at 37°C. The cells were washed twice with phosphate-buffered saline and subsequently fixed with 4% paraformaldehyde overnight. Then, the cells were resuspended in 500 μL of cell-penetrating fluid for 2 min at 0°C and treated with DNA denaturation working fluid for 30 min at 4°C. FITC-anti-BrdU (diluted 1:40) was mixed into the cell plate and incubated with the cells for 30 min at room temperature. The percentage of BrdU-positive cells was determined using a Flow Cytometer (BD, USA).

### Xenograft model

The animal procedures were approved by the Institutional Animal Care and Use Committee of Zhejiang Chinese Medical University (IACUC-20190909-02). All the female BALB/c nude mice used in this study were obtained from Shanghai National Laboratory Animal Center (Shanghai, China). Each 4-week-old nude mouse was subcutaneously injected with 5 × 10^6^ of the indicated cells suspended in 100 μL of phosphate-buffered saline (*n* = 6/group) or intraperitoneally transplanted with 8 × 10^6^ cells suspended in 150 μL phosphate-buffered saline (*n* = 5/group). The mice were sacrificed after 42 days, and their tumors were processed for hematoxylin and eosin staining and Ki-67 detection. The tumor volume was determined as follows: Volume = (length × width^2^)/2.

### Luciferase activity assay

We used pmirGLO dual-luciferase vector (Promega, Madison, WI, USA) to construct different luciferase reporter vectors. We co-transfected 293T cells with 50 ng of the corresponding luciferase reporter vector, 50 nm miRNA mimics and 5 ng of a Renilla luciferase reporter vector (pRL-TK, Promega) for 24 h. Luciferase activity was assessed with a dual luciferase reporter assay kit (Promega, E2920). The relative firefly luciferase activity was then normalized to the corresponding Renilla luciferase activity.

### RIP

The RIP experiment was performed with an EZ-Magna RIP Kit (Millipore, Billerica, MA, USA) according to the manufacturer’s protocol. An anti-AGO2 antibody (Abcam) was used for the RIP. The purified RNA was confirmed using qRT-PCR analysis.

### Fluorescence *in situ* hybridization

Cy3-labeled probe sequences for circSPIDR (5′-CY3-AUAGAACUUGGCUGAAAGUGUCUUUUGGUAAG-3′) and FITC-labeled probe sequences for hsa-miR-431-5p (5′-FITC-UGCAUGACGGCCUGCAAGACA-3′) were synthesized by Sangon Biotech (Shanghai, China) and used to analyze the co-localization of circSPIDR and miR-431-5p in CADC cells. Hybridization was performed overnight using the circSPIDR and miR-431-5p probes, and images were acquired using a Nikon inverted fluorescence microscope (Olympus).

### Western blotting

Total protein were extracted from treated HeLa cells. Then, equal amounts of protein were electrophoretically separated on a 8% sodium dodecyl sulfate polyacrylamide gel, and transferred to a polyvinylidene difluoride membrane. The membrane was blocked for nonspecific binding, incubated overnight with antibodies against SORCS1 (1:1000, Proteintech, 23002-1-AP), CUBN (1:500, Abcam, ab191073), or GAPDH (1:5000, Santa Cruz, sc-47724), and then incubated with a secondary antibody for 1 h. The bands of proteins were detected using SuperSignal West Pico Chemiluminescent Substrate (Pierce).

### Statistical analysis

All experiments were performed at least three times independently, and images from one representative experiment are shown. Statistical analyses were carried out using SPSS version 24.0 or GraphPad Prism version 9.0. The results are presented as the mean ± standard deviation. Differences between two groups were evaluated using a two-tailed Student’s *t* test. The correlations among circSPIDR, miR-431-5p, *SORCS1* and *CUBN* levels in CADC patients were assessed using Pearson’s correlation analysis. *P*-values ≤ 0.05 were defined as significant.

## Supplementary Materials

Supplementary Figures

Supplementary Table 1
