# Supplementary material for: CircSPIDR acts as a tumour suppressor in cervical adenocarcinoma by sponging miR-431-5p and regulating SORCS1 and CUBN expression
Source: Aging (Albany NY). 2021 Jul 29;13(14):18340–59. doi: 10.18632/aging.203283 (PMC8351706; doi:10.18632/aging.203283)
Supplement: Supplementary Figures [file aging-13-203283-s001.pdf]

## SUPPLEMENTARY FIGURES

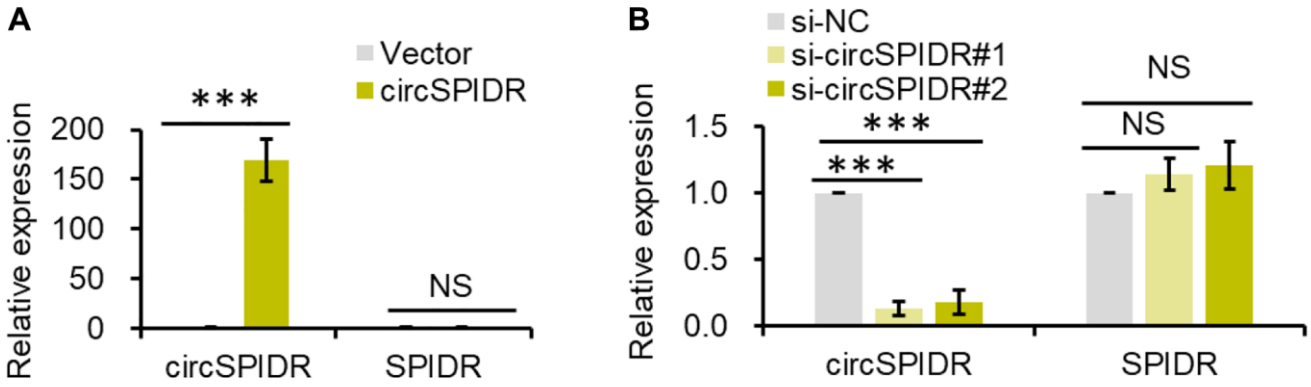


**Supplementary Figure 1. Transfection efficiancy of circSPIDR expression vector and siRNAs in HeLa cells.** (**A**) The expression level of circSPIDR in HeLa cells after transfection with circSPIDR expression vector was detected by qRT-PCR. Vector was taken as empty vector control. (**B**) The expression level of circSPIDR in HeLa cells after transfection with circSPIDR siRNA#1 or #2 was detected by qRT-PCR.si-NC was taken as negative siRNA control. NS, not significant; $^{***}P < 0.001$.

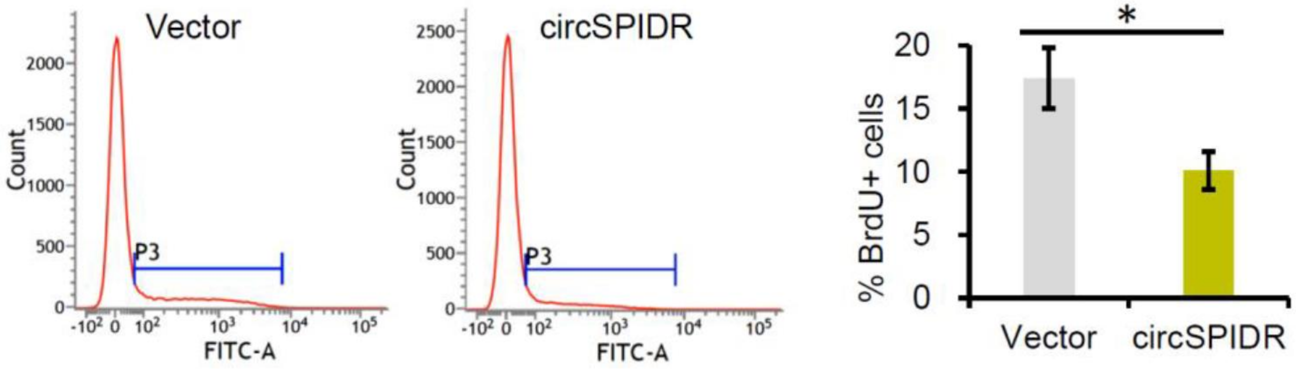


**Supplementary Figure 2. Overexpression of circSPIDR suppressed cell proliferation in HeLa cells by BrdU assay.** The percentage of BrdU positive cells was shown. $^{*}P < 0.05$.

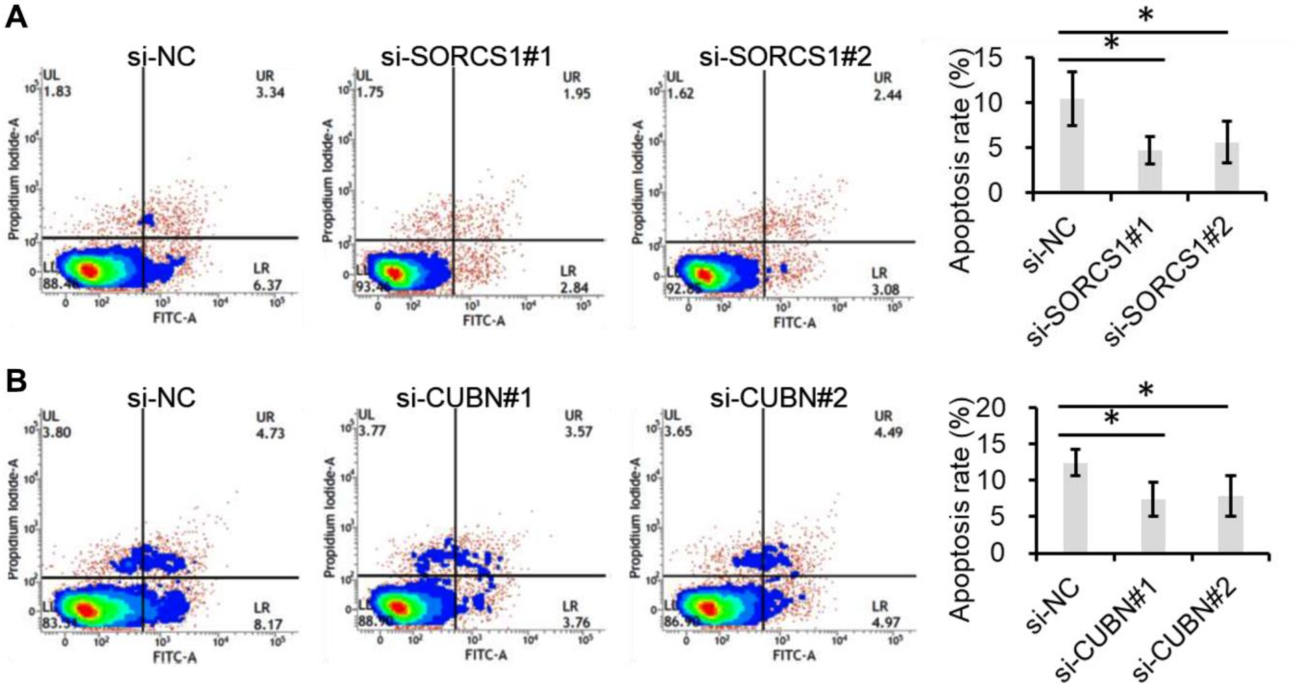


**Supplementary Figure 3. Knockdown of SORCS1 and CUBN decreased cell apoptosis in HeLa cells.** (**A**) Cell apoptosis assay for HeLa cells with SORCS1 knockdown. (**B**) Cell apoptosis assay for HeLa cells with CUBN knockdown. $^{*}P < 0.05$.

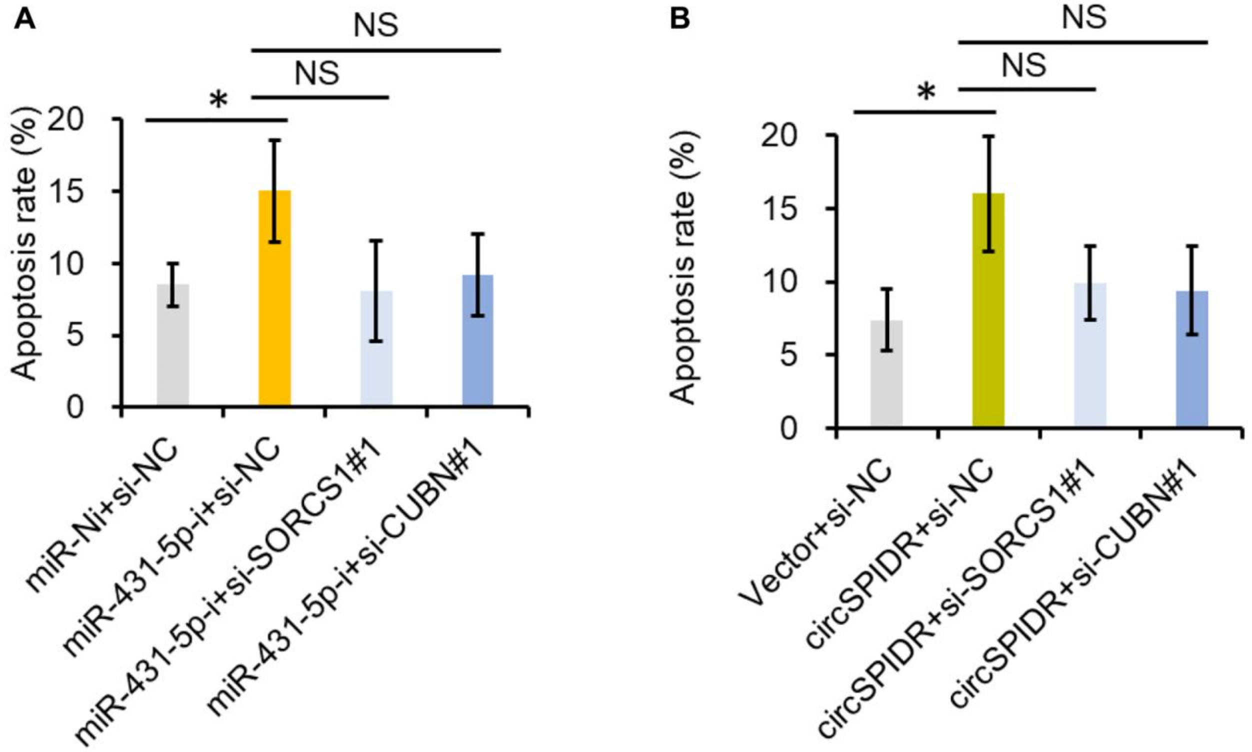


**Supplementary Figure 4. miR-431-5p and circSPIDR contribute to cell apoptosis via targeting SORCS1 and CUBN**. (**A**) Cell apoptosis assay for HeLa cells with miR-431-5p inhibitors and SORCS1 knockdown or CUBN knockdown. (**B**) Cell apoptosis assay for HeLa cells with circSPIDR and SORCS1 knockdown or CUBN knockdown. NS, not significant; $^{*}P < 0.05$.
