# Supplementary material for: CircSPIDR acts as a tumour suppressor in cervical adenocarcinoma by sponging miR-431-5p and regulating SORCS1 and CUBN expression
Source: Aging (Albany NY). 2021 Jul 29;13(14):18340–59. doi: 10.18632/aging.203283 (PMC8351706; doi:10.18632/aging.203283)
Supplement: Supplementary Table 1 [file aging-13-203283-s002.pdf]

SUPPLEMENTARY TABLE

Supplementary Table 1. The primer sequences used in this study.

Primers	Strand	Sequences（5′–3′）
circSPIDR	Forward	AAGACACTTTCAGCCAAGTTC
(divergent primers)	Reverse	CTCTCCATAATCTGTCTTGC
circSPIDR	Forward	ATTCAGAATCCCCTCACA
(convergent primers)	Reverse	CTGTTGATTTTGTCCTTG
SPIDR	Forward	ACTGGTGTGATCGTGTCTCTG
	Reverse	CGTGCACATTACCCAGCCTA
ADD2	Forward	CTCATTTCCTCCCAGCCTCG
	Reverse	GCCGCAGTTCCTTGACAAAA
CDHR1	Forward	TAATTGGTCTCGTCAGGCGG
	Reverse	GGGAGGGCAGAGACCACAAT
CUBN	Forward	AGGAACTGAGGAGGTGGACA
	Reverse	GACTGGAAGACGGCAGTGAA
DIRAS2	Forward	GAAGCTCTGAGCGGAGTTGT
	Reverse	AAACCTCAACACCAGGGAGC
EFCAB1	Forward	AGTAACTGAGACCTCACCGC
	Reverse	TGTCTGGCGCTCAGAGAATC
KLF8	Forward	TGTGGTATCTCCTCGTGGGT
	Reverse	GGTGGGGGCTATGAAAACCA
L3MBTL4	Forward	GTAGCTCGGTCGGCGTTG
	Reverse	TTGGGCTGTTTCATTGCCAC
NXPH3	Forward	GACCCCGAAAAGAGAAGGGG
	Reverse	ATAGAGGCTACCCTGCACCA
PLEKHG7	Forward	AAATTACCAGCTTCAGGGGCT
	Reverse	TGTGAAAAGTTCCCACACGG
SCN3B	Forward	GATTCCAGTCGGAACGCAAC
	Reverse	GGGTAAGCTCAGCTCGGAAG
SHE	Forward	TGTGACCCTCAGGAAGGTTAAG
	Reverse	TCCTCTTCCAGTCTGCACCA
SORCS1	Forward	CTGCCGGCATTTTGGTTCAA
	Reverse	ACCACCTTCCTGTACCCAGT
SOX5	Forward	AGGTAGCCATGGTGACAAGC
	Reverse	ACAAGTCTCTTGCGTCAGCA
TMEM213	Forward	CTCAACGTGGACTTCTGCCCA
	Reverse	CATCAGTTTGTCCACACAGAGC
WASF3	Forward	TGAGCCAAAGTGGTGATGCT
	Reverse	CGGGGCTCAATGTTCCTCTT
ZNF483	Forward	GCTCTACGCAATCCTAGCCA
	Reverse	AGTTATGTGCTCCACACAGCA
si-circSPIDR#1	Forward	AAAAGACACUUUCAGCCAATT
	Reverse	UUGGCUGAAAGUGUCUUUUTT
si-circSPIDR#2	Forward	CACUUUCAGCCAAGUUCUATT
	Reverse	UAGAACUUGGCUGAAAGUGTT
si-SORCS1#1	Forward	CCAACAAGCGUAAGAUAAUTT
	Reverse	AUUAUCUUACGCUUGUUGGTT

si-SORCS1#2	Forward	GCUGCCCUAUUGCUCACUATT
	Reverse	UAGUGAGCAAUAGGGCAGCTT
si-CUBN#1	Forward	GGAAUAUUCUAUGGCUCAATT
	Reverse	UUGAGCCAUAGAAUAUUCCTT
si-CUBN#2	Forward	GCAGACUACCCAAAUGAUATT
	Reverse	UAUCAUUUGGGUAGUCUGCTT
